# Management of adult patients with Langerhans cell histiocytosis: recommendations from an expert panel on behalf of Euro-Histio-Net

**DOI:** 10.1186/1750-1172-8-72

**Published:** 2013-05-14

**Authors:** Michael Girschikofsky, Maurizio Arico, Diego Castillo, Anthony Chu, Claus Doberauer, Joachim Fichter, Julien Haroche, Gregory A Kaltsas, Polyzois Makras, Angelo V Marzano, Mathilde de Menthon, Oliver Micke, Emanuela Passoni, Heinrich M Seegenschmiedt, Abdellatif Tazi, Kenneth L McClain

**Affiliations:** 1 Department of Medicine I, Center of Hematology an Stem Cell Transplantation, Hemostasis and Medical Oncology Internal Medicine I, Elisabethinen Hospital, Fadinger Str. 1 4010, Linz, Austria; 2 Department of Pediatric Hematology Oncology, Azienda Ospedaliero Universitaria A. Meyer, Florence, Italy; 3 Departament of Respiratory Medicine, Hospital de la Santa Creu i Sant Pau, Barcelona, Spain; 4 Imperial NHS Trust, London, UK; 5 Clinic for Internal Medicine, Protestant Clinics, Gelsenkirchen, Germany; 6 Paracelsus Klinik, Osnabrück, Germany; 7 Service de Medicine Interne, Groupe Hospitalier Pitie-Salpetiere, Paris, France; 8 Department of Pathophysiology, University of Athens School of Medicine, Athens, Greece; 9 Department of Endocrinology and Diabetes, 251 Hellenic Air Force & VA General Hospital, Athens, Greece; 10 U.O. Dermatologia, Fondazione IRCCS Ca´ Granda-Ospedale Maggiore Policlinico, Milano, Italy; 11 Department of Internal Medicine, Hospital Saint Louis, Paris, France; 12 Department of Radiotherapy and Radiation Oncology, Franziskus Hospital, Bielefeld, Germany; 13 Radiation Oncology Center, Hamburg, Germany; 14 Pulmonolgy Department, Saint Louis Teaching Hospital, Paris, France; 15 Texas Children’s Cancer Center/Hematology Service, Houston, TX, USA

**Keywords:** Langerhans, Adult, Histiocytosis

## Abstract

Langerhans Cell Histiocytosis (LCH) is an orphan disease of clonal dendritic cells which may affect any organ of the body. Most of the knowledge about the diagnosis and therapy is based on pedriatic studies. Adult LCH patients are often evaluated by physicians who focus on only the most obviously affected organ without sufficient evaluation of other systems, resulting in patients being underdiagnosed and/or incompletely staged. Furthermore they may be treated with pediatric-based therapies which are less effective and sometimes more toxic for adults. The published literature on adult LCH cases lacks a comprehensive discussion on the differences between pediatric and adult patients and there are no recommendations for evaluation and comparative therapies. In order to fill this void, a number of experts in this field cooperated to develop the first recommendations for management of adult patients with LCH. Key questions were selected according to the clinical relevance focusing on diagnostic work up, therapy, and follow up. Based on the available literature up to December 2012, recommendations were established, drafts were commented by the entire group, and redrafted by the executive editor. The quality of evidence of the recommendations is predominantly attributed to the level of expert opinion. Final agreement was by consensus.

## Background, process of development and restrictions

There are no universally accepted international guidelines available for the diagnosis and treatment of adult LCH patients. The largest number of patients was published in a pooled retrospective analysis from several national registries [1].

Based on the available literature up to December 2012 and personal experience the following recommendations were established by an international group of academic clinicians who are recognized experts in the field of histiocytic disorders. Grading of recommendations based on levels of evidence and agreement between experts is listed in Table 1.

**Table 1:** Grade of recommendation

**Level of evidence**	**Level of agreement between experts**	
**A** meta-analyses, high quality systematic reviews, or randomized controlled trials	**2** general agreement between all experts	
**B** systematic reviews of case control or cohort studies	**1** discussed recommendation, but no formal objections between experts	
**C** non-analytic studies: for example, case reports, case series, small retrospective studies	**0** divergence of opinion	
**D** expert opinion		

Due to the diversity of clinical course of LCH, even recommendations which are established as standard of care may need to be critically appraised in an individual case and involvement of a LCH expert should be considered. A map of experts, reference centers and additional information about the disease is available on the web-site of Euro-Histio-Net (http://www.eurohistio.net) and the Histiocytosis Association (https://www.histio.org/).

### General consideration

The etiology of LCH is unknown. LCH cells are clonal (except primary pulmonary LCH) [2,3] and a cancer-associated mutation (*BRAFV600E)* was found in more than half of investigated specimens, indicating that LCH may be more a neoplastic (not a malignant!) disease than a reactive disorder, but the pathogenesis is still unclear [4,5]. Although apparent associations between LCH and malignant tumors have been recognized, these cases represent a minority of all LCH patients and the pathophysiologic relationship remains undefined [6].

The disease may affect any organ or system, more frequently bones, skin, and pituitary gland. Lymph nodes, liver, spleen, gut, the central nervous system, pituitary, and the hematopoietic system are less frequently affected. Lungs may be affected simultaneously or consecutively with other organs, but isolated pulmonary LCH (PLCH) occurs frequently in adults and may proceed to multisystem involvement. PLCH requires a different management in contrast to multi-organ involvement and is therefore discussed in a separate section.

Clinical manifestations of LCH vary depending on the organ or system affected, from self-healing disease to chronic recurrences. A rapid progressive form, seen in children, is usually not observed in adults. Langerhans cell sarcoma (malignant histiocytosis) can occur de novo or from an antecedent LCH [7]. This paper will not cover other histiocytic disorders such as Erdheim-Chester disease (ECD), Rosai-Dorfman disease (RDD) or malignant histiocytosis. In cases of occurrence of LCH and ECD or RDD in the same patient, the management is based on the predominant disease.

**Figure 1:**
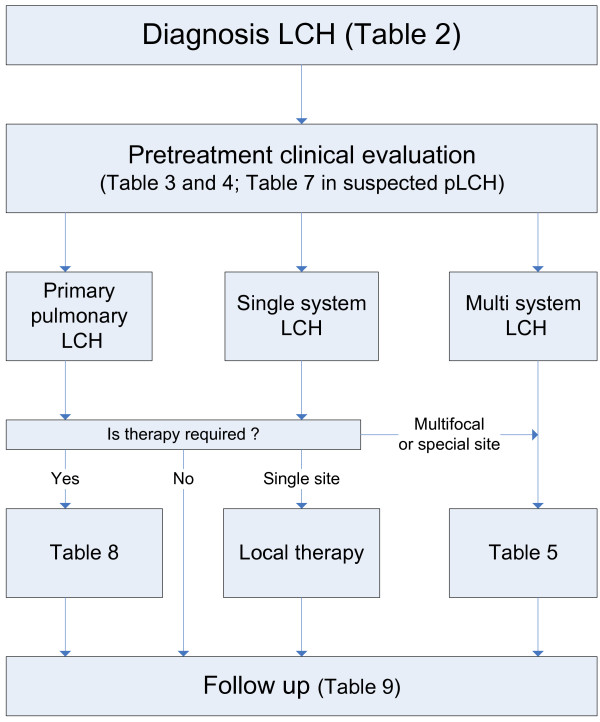
Management of Langerhans Cell Histiocytosis in adults.

Treatment options vary depending on disease extent and severity at onset. A uniform diagnostic work-up is necessary (see Figure 1). One of the main problems of LCH in adults is the variety of potentially involved organs resulting in several physicians being consulted. Frequently only the most obviously affected site is considered and a complete examination is not done thus missing other sites of disease.

### Diagnosis

The diagnosis of LCH should be based on histologic and immunophenotypic examination of a lesional biopsy. Normal Langerhans cells stain positively with CD1a and/or Langerin [8-10]. Misdiagnoses of LCH have occurred, as the presence of normal reactive LCs in skin and regional lymph nodes may be confusing.

The two levels of certainty of LCH diagnosis which are generally agreed upon are shown in Table 2[11].

**Table 2:** Diagnostic criteria of LCH

**Definitive:**	**Presumptive (or compatible):**
Based on clinic-pathological evidence with microscopic examination and at least one of the following immunological staining:	Based only on clinico-radiological evidence, without biopsy, as in case of:
• Langerin (CD 207) positivity	e.g.: Pulmonary lesions on CT scan with typical cysts and nodules in a smoker. (however, biopsy should be considered in order to reach a more definitive diagnosis)
• CD1a positivity	
• Presence of Birbeck granules on electronic microscopy	

### Pretreatment clinical evaluation

#### Complete history

Patients with LCH are often asymptomatic or show only mild symptoms. The most common symptoms are dyspnea, cough, bone pain, an abnormal growth of soft tissue over the affected bone, rash, pruritus, increased thirst, and lymphadenopathy. Additional signs are fatigue, generalized weakness, weight loss, night sweats, nausea, and fever.

A thorough history should be performed including the questioning about unexplained symptoms in the past such as “idiopathic” eczema, thyroid disease or diabetes insipidus, lung cysts or pneumothorax, or bony lesions, the smoking and family history with special attention to autoimmune disease. A very small number of familial cases are reported [12].

#### Complete physical examination

A comprehensive physical examination is necessary. The skin and visible mucous membranes should be inspected. Supplemental neurological and/or psychological investigations are useful in patients presenting with neuromyopathy or cognitive impairment.

#### Laboratory and radiographic evaluation

The laboratory tests to be performed for all patients independently of affected organs include a complete blood count, blood chemistry, coagulation studies, thyroid stimulating hormone (TSH), freeT4 and urine analysis - see Table 3 (Grade D2).

**Table 3:** Baseline laboratory and radiographic evaluation

**Recommendation**	**Grade**
Full Blood Count (Hemoglobin, White blood cell and differential count, Platelet count)	D2
Blood Chemistry (Total protein, Albumin, Bilirubin , ALT (SGPT), AST (SGOT)	D2
Alkaline phosphatase (AP), gammaglutamyl transpeptidase (γGT)	
Creatinine, Electrolytes, CRP (C-reactive Protein)	
Erythrocyte Sedimentation Rate (ESR)	D1
Coagulation Studies (INR/PT, Fibrinogen)	D2
Thyroid Stimulating Hormone (TSH), freeT4	D2
Morning Urine Osmolarity	D1
Urine Test Strip	D2
Ultrasound (liver, spleen, lymph-nodes, thyroid gland)	D2
Chest Radiograph (CXR)	D2
Low Dose Whole Body (Bone) CT (if not available: X-Ray Skeletal/Scull Survey)	D2
Optional: Baseline Head-MRI	D2
Optional: PET-CT instead of Ultrasound, CXR and Bone CT	D2

A skeletal survey, skull series (or low dose whole bone CT [13]) and chest x-ray (AP and lateral) are the first radiographic examinations to be done. CT of specific areas of the skeleton are indicated when mastoid, orbital, scapular, vertebral, or pelvic lesions are found by plain x-rays. MRI may detect additional osseous or extraosseous lesions. A skeletal scintigram (bone scan) alone does not suffice.

Any evidence of a pathological thoracic finding should be followed up by high-resolution chest CT. Ultrasonographic examination of the abdomen may reveal hepatic abnormalities. An ultrasound of the neck with attention to the thyroid gland may be indicated if there are thyroid nodules or evidence of thyroid dysfunction. A MRI of head is needed for hypothalamic/pituitary or brain abnormalities. PET-(CT) scan may identify lesions missed by other modalities and documents response to therapy [14].

Further investigations may be indicated based on the patient's symptoms and the findings of the basic diagnostic tests - see Table 3 and 4 (Grade D2).

**Table 4:** Specific clinical scenarios: recommended additional testing

**Recommendation**	**Grade**
**History of polyuria or polydipsia:**	D2
• Urine and Plasma osmolality	
• Water deprivation test	
• MRI of the head	
**Suspected Other Endocrine Abnormality:**	D2
• Endocrine assessment (including dynamic tests of the anterior pituitary, MRI of the head)	
**Bi- or Pancytopenia, Or Persistent Unexplained Single Cytopenia:**	D2
• Any other cause of cytopenia has to be ruled out according to standard medical practice	
• Bone marrow aspirate and trephine biopsy to exclude causes other than LCH	
• In case of morphological signs of hemophagocytosis additional tests like serum-ferritin should be performed (criteria of HLH)	
**Liver Or Spleen Abnormalities:**	D2
• In case of any unclear sonographically pathology CT, PET-CT, MRI or Scans should be added (the choice is depending on the sonomorphology – discuss with your radiologist)	
• Visuable lesions of the liver should be biopsied if possible	
• Other causes of splenomegaly has to be ruled out before it may be assigned to LCH	
• ERCP (Endoscopic Retrograde Cholangiopancreatography) or MRCP (Magnetic Resonance Cholangiopancreatography) should be performed in case of elevated serum cholestasis markers or sonomorphologically dilatated bile ducts. Primary biliary cirrhosis and primary sclerosing cholangitis have to be ruled out.	
**Unexplained Chronic Diarrhea, Weight loss, Evidence Of Malabsorption Or Hematochezia**	D2
• GI-Exploration (Endoscopy with biopsies, capsule endoscopy)	
**Enlarged Lymph Nodes (LN):**	D2
• If found by screening ultrasound or physical examination the best suitable LN should be extirpated. A LN needle biopsy should be avoided.	
• CT scans or a PET-CT should be performed additionally	
**Lung Involvement - In case of abnormal Chest X Ray or symptoms/signs suggestive for lung involvement or suspicion of a pulmonary infection:**	D2
• Lung high resolution computed tomography (HR-CT)	
• Lung function tests (Spirometry, Diffusing capacity, Oxygen desaturation during exercise (6MWT), blood gases)	
• Bronchoalveolar lavage (BAL): > 5% CD1a + cells in BAL fluid may be diagnostic of LCH	
• Lung biopsy (if BAL is not diagnostic), ideally Video-assisted thoracoscopic surgery (VATS)	
**Osseous Disease:**	D2
• CT +/- MRI should be performed in case of craniofacial or vertebral lesions or signs of additional soft tissue involvement	
• Biopsies should be taken from the most suitable region in case of multifocal bone disease	
**Skin, Oral And Genital Mucosa lesions:**	D2
• Biopsies should be taken	
**Aural Discharge Or Suspected Hearing Impairment / Mastoid Involvement:**	D2
• Formal hearing assessment	
• MRI of head	

### Definition of organ involvement

#### Possibly involved organs

After the diagnosis of LCH has been made, involvement of other organs should be evaluated and defined according to the clinical, biological or radiological criteria.

### Risk organs (bone marrow, liver, spleen, CNS)

Involvement in the hematopoietic system (extremely rare in adults), spleen, liver or CNS indicates a less favorable prognosis, with possible mortality if the patient does not respond to therapy. Although this has never been proven for adults, retrospective analyses of national registries and the experts’ experience support the existence of the above mentioned “risk organs”.

Fever, night-sweats and weight loss combined with poor performance score might predict the rarely observed aggressive course of LCH in adults comparable to that of high grade non-Hodgkin lymphoma [15,16].

### “Special Sites” and “CNS-Risk” bone involvement

Vertebral lesions with intraspinal or cranofacial bone lesions with soft tissue extensions (orbit, mastoid, sphenoid or temporal bones) may cause immediate risk to the patient because of the critical anatomical site and the hazards of attempting local therapy. Isolated disease in these “Special Sites” justifies systemic therapy for children because of spinal cord compression and the association of cranio-facial bone lesions with an increased risk of developing diabetes insipidus [17]. It is unclear if this connection might be extrapolated to adults, but most experts treating LCH patient follow the same guidelines for their adult patients as with the pediatric cases (Grade D2).

### Endocrinologic dysfunction

LCH exhibits a predilection for the hypothalamo-pituitary (HP) region leading to permanent posterior and/or anterior pituitary hormonal deficiencies in a subset of patients.

Diabetes Insipidus (DI) is the most common disease-related consequence that can predate the diagnosis or develop anytime during the course of the disease [18,19]. DI is found in up to 30% of patients [1], but may reach to 40% in patients with multisystem disease or 94% in the presence of other pituitary deficiencies [18,20]. Polyuria and polydipsia, and/or structural abnormalities of the HP region dictate investigations to confirm DI.

Anterior pituitary dysfunction (APD) is found in up to 20% of patients, almost always with DI [18,21]. Although APD is not invariably associated with abnormal HP imaging it is almost always encountered in patients with MS LCH who have DI and HP pathology on MR imaging [22]. Growth hormone deficiency (GHD) is the most frequent disease-related APD found in up to 50% of patients with DI [20]. In adults there are no specific GHD-related symptoms that can suggest the diagnosis [23]. Gonadotropin deficiency is the second most common deficiency, presenting with menstrual disturbances in women and decreased libido in men [20]. ACTH deficiency may be partial or complete and present either with non-specific symptoms or as acute adrenal insufficiency following stressful events. TSH deficiency is almost always associated with panhypopituitarism and may present with subtle symptoms or obvious signs of hypothyroidism. Moderately elevated prolactin levels attributed to pituitary stalk infiltration can cause galactorrhoea in females and gonadotropin deficiency in all patients. Established endocrine deficiencies almost never recover over time, although apparent HP abnormal imaging may often regress either in response to treatment or as a result of the “natural course” of the disease [22].

Hypothalamic involvement is less frequent than pituitary involvement and leads to not only pituitary dysfunction, but neuropsychiatric and behavioral disorders, disturbances of thermo-regulation and sleeping pattern, and autonomic and metabolic abnormalities. The most frequent consequence is severe obesity due to increased appetite. Hypothalamic-related adipsia may seriously complicate the management of DI.

Metabolic abnormalities: One study involving 14 adult patients and 42 controls has shown that adults with LCH are at high risk of developing abnormalities of carbohydrate metabolism (diabetes mellitus, impaired glucose tolerance) and lipid metabolism leading to increased insulin resistance even in the absence of obesity [24].

Bone metabolism: Adults with LCH may present with a lower than expected bone mineral density at any age especially during periods of active disease [25].

Investigation of hormonal deficiencies: Evaluation of TSH, free T4 and morning urine osmolality is recommended in all patients, further procedures (water deprivation test, plasma osmolality, serum cortisol, insulin like growth factor I, gonadal steroids and gonadotropin serum levels) to detect partial DI or anterior pituitary deficiencies should be performed when clinical symptoms are present (Grade D2).

#### Dermatological involvement

Cutaneous LCH can be the great pretender, mimicking a number of common dermatoses, and may represent the earliest sign of the disease [26]. The typical scalp lesions are small translucent papules, 1-2 mm in diameter, slightly raised and rose-yellow in colour. These lesions frequently show scaling or crusting, often leading to a misdiagnosis of seborrheic dermatitis.

Intertriginous involvement in the axillary, inguinal, vulvar, or anogenital regions with erythema and erosions are frequently misdiagnosed as eczema, psoriasis, Candida infection, or intertrigo. Generalised skin eruptions can mimic guttate psoriasis prurigo nodularis or lichen planus.

Gingival involvement is frequently associated with alveolar bone involvement and loosening of teeth. Tooth extraction should be avoided as with treatment they will embed into the recovering alveolar bone. Nail changes include paronychia, onycholysis, subungueal hyperkeratosis and purpuric striae of the nail bed, suggesting a wide panel of conditions. Dark-brown striae similar to those drug-induced are also seen.

Cutaneous LCH has so many different manifestations that one needs a high level of suspicion and biopsy is essential. Although skin disease may be the primary presentation, one must investigate for systemic disease (Grade D2).

#### Gastrointestinal involvement

Gastrointestinal (GI) tract involvement by LCH is rare and may appear as a solitary colorectal polyp or multiple granulomatous lesions of the mucous membrane in the upper and lower GI tract [27]. Patients are often asymptomatic. Multiple infiltrations are associated with abdominal pain, diarrhea, and hypoalbuinemia.

Liver infiltration is characterized sometimes by infiltration of CD1a+ cells in nodules or by lymphocytes alone along the portal tracts which may lead to sclerosing cholangitis. In case of splenomegaly other causes than LCH primarily have to be ruled out. Pancreatic involvement (mainly tumorous) is extremely rare.

### Stratification

Single System LCH (SS-LCH): One organ/system involved (uni- or multifocal):

➢ Bone: unifocal (single bone) or multifocal (> 1 bone)

➢ Skin

➢ Lymph node

➢ Hypothalamic-pituitary / Central nervous system

➢ Lungs (primary pulmonary LCH)

➢ Other (e.g. thyroid, gut)

Multisystem LCH (MS-LCH): Two or more organs/systems involved:

➢ With involvement of “Risk Organs” (Hematopoietic system, spleen, and/or liver, tumorous CNS)

➢ Without involvement of “Risk Organs”

### Treatment

#### Management algorithms (see Figure 1)

Treatment recommendations are based on site and extension of the disease.

#### Careful observation, local or “mild systemic” therapy

##### Bone involvement

In case of single system LCH with unifocal bone involvement of “non-CNS-Risk facial bones” local therapy and careful observation is recommended. The modality of treatment depends on location, size, and symptoms of the disease. Biopsy or curettage is suitable for histopathologic diagnosis and initiating a healing process. Complete excision of bone lesions is not indicated as it may increase the size of the bony defect and the time to healing or result in permanent skeletal defects. Intralesional injection of steroid may hasten healing. Dosages of 40 – 160 mg of methylprednisolone have been used [28] (Grade C2). Radiotherapy is indicated if there is an impending neurological deficit and a high surgical risk, e.g. lesion in the odontoid peg or cranial base. For multifocal bone LCH and for bone lesions in “special sites” systemic therapy (see next page under front line treatment) should be given (Grade D2).

##### Isolated lymph nodes involvement

Isolated lymph nodes involvement is rare but spontaneous regressions have been observed. Thus extensive surgery (e.g. neck-dissection) and systemic therapy should be omitted [29] (Grade C2).

##### Skin Involvement

Surgical excision should be limited to solitary lesions, but mutilating surgeries such as hemi-vulvectomy should not be performed (Grade D2). If the patient is being treated for multisystem disease the skin will respond to treatment. In single system skin disease or in the rare instance where the skin fails to respond fully to systemic treatment for multisystem disease there are a number of treatments directed specifically to the skin.

Topical nitrogen mustard: 20% nitrogen mustard applied to the skin is an effective treatment in children [30]. There is no published data on treatment in adults and there are problems with availability in most countries (Grade C1).

Phototherapy: Psoralen plus ultraviolet A (PUVA) [31] and narrow band ultraviolet (UV) B [32] are effective in treating cutaneous LCH in individual case reports. It is difficult to treat patients with intertriginous or scalp involvement and would be contraindicated in penile disease (Grade C1).

Thalidomide: is a TNF-α antagonist and has been shown to be effective in treating cutaneous LCH [33] but gives poor responses in high risk multisystem disease [34]. Dose of 100mg/day in adults is generally used but toxicity with peripheral neuropathy must be monitored (Grade C2).

Azathioprine: There are no published reports of the use of azathioprine (or its metabolite 6-mercaptopurine) in adults with cutaneous LCH but it is a useful drug in single system skin as well as multisystem disease [35]. Patients need to be tested for thiopurine methyl transferase, and if normal should be treated at a dose of 2mg/kg/day. The drug takes about 6 weeks to become effective (Grade D1).

Methotrexate: There are published reports on the use of low dose methotrexate as either single agent treatment or in combination with azathioprine or prednisolone. Methotrexate was used successfully at the dosages of 20mg once weekly [36] (Grade C1).

##### Involvement of the oral mucous membranes

These lesions should be treated with “mild systemic” therapy as described above and extraction of teeth should be avoided as much as possible. In refractory cases more intensive systemic treatment is required (see next paragraph) (Grade D2).

#### Systemic therapy

##### Front line treatment

Systemic therapy should be considered in case of the following disease category:

➢ MS-LCH with/without involvement of “risk organs”

➢ SS-LCH with multifocal lesions

➢ SS-LCH with “special site” lesions

There is no standard first line therapy like in pediatric LCH. Vinblastine/prednisolone is mentioned in various chemotherapeutic manuals, but has never been proven effective for adults in a prospective study. An international trial failed because of low recruitment rate. Due to lower risk of neurotoxicity and frequently observed unacceptable steroid induced side effects some experts prefer monotherapy with cladribine, cytarabine or etoposide [35]. In a retrospective study evaluating 58 adult patients with bone lesions the authors observed a clear superiority of cytarabine especially to vinblastine/prednisolone but even to 2-CDA in terms of response and toxicity [37]. Intensive combination chemotherapies (e.g. MACOP-B) are effective [38] but should be used only in rare cases of an aggressive LCH form [15] (Grade C1).

Until recently, most experts started with 2-CDA in case of risk organ or tumorous cerebral involvement, but cytarabine may be a reasonable alternative (Grade C2).

Some investigators have used bisphosphonates for multifocal bone disease, but patients have to be advised to the risk of osteonecrosis of the jaw and its prevention [39]. COX-Inhibitors might be more than analgetic drugs and regression of LCH was observed [40] (Grade C2).

Grade of recommended systemic first line therapy is listed in Table 5.

**Table 5:** First line systemic therapy

**Recommendation**	**Grade**
**Mild Symptoms, No Risk Organ Involved:**	
• Methotrexate 20 mg per week p.o/i.v.	C1
• Azathioprine 2 mg/kg/d p.o	D1
• Thalidomide 100mg/d p.o in skin or soft tissue multifocal single system LCH	C2
**Additionally In Multifocal Bone LCH**	
• zoledronic acid 4 mg i.v.	C2
q 1 (- 6) month (depending on extent and response)	C1
**Symptomatic, MS-LCH, No Risk Organs involved**	
• Cytarabine 100 mg/m^2^ d1-5 q4w i.v.	C1
• Etoposide 100 mg/m^2^ d1-5 q4w i.v.	D1
• Vinblastin/Prednisolone (like in pediatric studies)	C1
**MS-LCH, Risc Organs Involved**	
• 2-CDA 6 mg/m^2^ d1-5 q4w s.c./i.v.	C2

##### Evaluation of response

Evaluation is done after 2 to 3 cycles of chemotherapy. If there is disease progression or reactivation, complete evaluation as recommended in the previous section has to be performed (Grade D2).

##### Maintenance therapy

Etoposide or 2-CDA are usually administered up to 6 months. Cytarabine can be given at low dose monthly up to a year in most patients (6-12 cycles) (Grade D2).

##### Salvage therapy

Refractory disease should be treated with drugs not used for the first course. In case of further progression, especially in CNS involvement cytarabine may be added to 2-CDA (both drugs cross the blood brain barrier) [41]. Some cases with response to tyrosine kinase inhibitors (imatinib) have been reported [42,43]. In the rare case of a most aggressive course of disease hematopoietic stem cell transplant has been performed successfully as well [44,45]. Clofarabine has been effective for refractory childhood LCH [46] (Grade C2).

### Treatment options in case of reactivation

Reactivations of LCH in adults occur in about 25-38% of the patients (European national registry data and [37]). Patients may have further reactivations especially those with multisystem disease.

#### Reactivation of single system disease

The choice of treatment options is based on the same principles as for initial disease.

The options for reactivations of SS-LCH (skin, bone, other) include

I. Wait and watch approach

II. Local therapy including irradiation (as above)

III. Bisphosphonates for bony disease (as above)

IV. Chemotherapy (as above)

In case of a multisystem reactivation of a SS-LCH, treatment should follow the options for MS-LCH including systemic therapy (Grade D2).

The efficacy of 2-CDA for single and multisystem reactivated LCH has been proved in a phase II trial [47].

#### Reactivation after systemic therapy

I. If the reactivation is more than one year after completion of treatment, re-induction with the prior chemotherapy may be effective. If however, the disease is not responsive we suggest discussion with the reference centre for your country.

II. If reactivation occurs while on treatment, potentially 2nd line strategies as described above, but should be generally discussed with your reference centre (Grade D2).

### Radiotherapy

In contrast to pediatric recommendations, radiotherapy is an effective treatment option with acceptable side-effects for adult patients with LCH in selected situations [48-52].

Most literature data concerning radiotherapy in adult LCH deal with uni- or multifocal osseous single-system disease. The local control rates ranged from 75-100%, complete remission from 79-100%, respectively [53].

The dose recommendation for radiotherapy is still controversial and an exact dose-effect relationship has not been established. There is a wide dose range of applied total doses from 1,4 Gy up to 45 Gy. In general, a dose range from 10 to 20 Gy is recommended in adults [50,54]) (Grade C2).

Recommended indications for the use of radiotherapy in adults with LCH are listened in Table 6.

**Table 6:** Possible indications for the use of radiotherapy in adults

**Recommendation**	**Grade**
**Isolated “Unresectable” lesion:**	C2
if a resection would significantly compromise anatomic function, e.g. odontoid peg, CNS	
**Recurrent or progressive lesion:**	C2
In multifocal or multisystem disease only in case of minor response to standard systemic therapy	
**Adjuvant treatment following marginal or incomplete resection:** especially in single system bone disease with soft tissue involvement	C2

### Treatment and hormone replacement of endocrinopathies

DI should be treated with desmopressin. The timing and dosage must be individualized. In proven LCH new onset DI is a sign of active disease and initiation of systemic treatment is recommended to try to prevent the development of further hormonal deficiencies although existing ones usually do not resolve [55]. Adequate replacement of hormonal deficiencies should be initiated as soon the diagnosis is made (Grade D2).

### Central nervous system involvement

#### Tumorous lesions

These lesions are most frequently observed in the hypothalamic-pituitary region. The tumor size ranges from discrete thickening of the pituitary stalk to larger tumors. Parenchymal, meningeal or choroid plexus lesions occur less frequently [56].

In addition to hormone replacement isolated cerebral tumors should be treated with irradiation or chemotherapy and pituitary/hypothalamic lesions with chemotherapy. Multifocal brain lesions or single brain lesions with multi system disease need to be treated with chemotherapy. The most suitable drugs are Cladribine or Cytarabine as described above (Grade C2).

#### Neurodegerative LCH

Non-tumorous MRI findings of the cerebellum, and/or brain stem are histopathologically different than the typical LCH mass lesions. The neurodegenerative lesions lack CD1a+ histiocytes and have infiltrating CD8+ lymphocytes [55]. Some of these patients show no symptoms, others have clinical signs ranging from subtile tremor, dysarthria, dysphagia, and motor spasticity to pronounced ataxia, behavioral disturbances and severe psychiatric disease.

Retinoic acid and intravenous immunoglobulin may stabilize such patients [57,58]. Improvement with infliximab has been observed in one case [59]. Cytarabine with or without Vincristine provided improvement in 5/8 patients of which 4/8 remained stable over more than 7 years of follow-up and one relapsed but is improved after treatment with intravenous Methotrexate. Patients who responded to Cytarabine/Vincristine had symptoms for less than 18 months before starting treatment [60,61]. Thus early onset of Cytarabine is recommended as first line therapy, but for any case of neurodegenerative LCH we suggest discussion with the reference centre for your country (Grade C1).

### Primary pulmonary LCH

#### Epidemiology

The incidence of pulmonary LCH (PLCH) is unknown. Reports provided by histopathological studies and interstitial lung diseases registries revealed about 5% of PLCH in this population [62]. Data from a Japanese survey show an estimated prevalence of 0.07-0.27/100000 population in females and males, respectively [63]. The prevalence may be underestimated.

PLCH affects mainly young, predominantly smoking (> 90%) adults with a peak at 20-40 years of age and a slight predominance of women. It is unknown if there are any racial differences in this disease [62].

### Clinical features

Patients with PLCH often present with a non-productive cough or dyspnoea, chest pain, associated non-specific symptoms like fatigue, weight loss, night sweats and fever may be observed [62,64]. About 20% of patients with PLCH are initially asymptomatic and an equal percentage of patients present with acute symptoms of a pneumothorax.

It is important to exclude the existence of multi system LCH. Thus, a thorough history, comprehensive physical examination, and baseline radiographic, blood and urine tests should be performed in any patient presenting with PLCH to avoid undertreatment. (see Table 3 and 4).

### Diagnosis

X-Ray of the chest shows a reticulo-micronodular pattern. In more advanced cases cysts may be visible within the infiltrates symmetrically in both lungs, but predominating in the middle and upper lung fields and sparing the costophrenic angles [62].

High resolution CT (HRCT) is the most important visualizing tool for PLCH [62]. The typical HRCT pattern is of small nodules, cavitated nodules (both may resolve), and thick- and finally thin-walled cysts. As the disease evolves, cystic lesions become a predominant finding.

Pulmonary lung function tests most frequently show reduced diffusing capacity of the lung for carbon monoxide (DLCO), 70–90% of the patients. Lung volumes are impaired in a majority of patients with decreased vital capacity and air trapping (elevated residual volume). Total lung capacity is within normal values in most cases. An obstructive pattern is observed in a sizeable proportion of patients, particularly in advanced disease. Rarely a restrictive component may appear [65]. A predominantly nodular pattern suggestive of active inflammatory disease can have only moderate functional consequences [65].

Bronchoalveolar lavage (BAL) often shows high alveolar macrophage counts, reflective of smoking. Infection should be systematically ruled out. BAL yielding more than 5% CD1-positive cells has previously reported to support the diagnosis of pulmonary LCH [66]. While this has a high specificity, BAL results lack sensitivity.

Bronchial biopsies are not helpful in the diagnosis of PLCH but are useful in ruling out other diagnoses in patients with atypical manifestations. The diagnostic method of choice is therefore videothoracoscopic lung biopsy after HRCT evaluation (see Table 7). In asympto-matic patients with a typical HRCT pattern and a macrophage alveolitis by BAL, for whom no systemic therapy is required, a presumptive diagnosis may be acceptable with a close follow-up. In patients with extensive cystic lesions, the risk of invasive procedures has to be balanced with the need for a definitive diagnosis (Grade D2).

**Table 7:** Diagnostic recommendations in pLCH

**Recommendation**	**Grade**
**Confirm definitive diagnosis**	
• in all patients before start of systemic therapy	D2
• prefer lung biopsy	D2
• HRCT is required in all patients	D2
**Exclude Existence Of Multi System LCH**	D2

### Treatment and prognosis

The natural history of adults with PLCH is widely variable and mostly unpredictable in the individual patient. About 40-50% of patients with PLCH experience a favorable outcome and partial or complete clearance of the radiological abnormalities occurs with or without therapy.

Serial lung function tests are essential for following patients with PLCH. In a recent retrospective multicenter study, lung function (mainly DLCO and FEV_1_) deteriorated in approximately 60% of the patients [65]. An isolated decline of DLCO in symptomatic patients should prompt a search for pulmonary hypertension by echocardiography and in case of increased systolic pulmonary arterial pressure should be confirmed by right heart catheterization [67].

Based on the epidemiologic data smoking cessation is essential. Patients with a stable disease despite ongoing smoking should be told about all other known medical reasons for ceasing smoking and enrollment in a support group may be valuable [62,64].

There are no study-based data supporting cortisone therapy for pulmonary LCH. Any possible therapeutic benefit for symptomatic patients should, therefore, be carefully weighed against the potential undesired effects of this form of treatment, because spontaneous remissions do occur. If smoking cessation failed and treatment is required systemic steroid therapy (usually 1mg/kg/day for one month, followed by tapering dosages over months) may be given in patients with the nodular form of pulmonary LCH [62,64].

Lower respiratory tract infection is a common cause of deterioration of PLCH and should lead to prompt treatment. Annual vaccination against influenza as well as anti-pneumococcal is recommended for patients with impaired lung function.

Progressive PLCH despite steroid therapy may be treated with 2-CDA [68,69]. A randomized controlled trial evaluating the effectiveness and tolerance of 2-CDA in this subgroup of patients is ongoing.

Pneumothorax requires drainage and pleurodesis should be considered in case of recurrence [70]. Lung transplantation (LT) may represent a therapeutic option in case of advanced PLCH (severe respiratory failure or major pulmonary hypertension). Recurrence of LCH after transplatation occurs in 20% without impact on the survival rate [71].

Grades of recommendations for therapy in pLCH are listed in Table 8.

**Table 8:** Therapeutic recommendations in pLCH

**Recommendation**	**Grade**
First step is smoking cessation in all patients	C2
Watchful waiting in a- or minor symptomatic patients	C2
Systemic steroid therapy in symptomatic patients	C2
Chemotherapy (e.g. 2-CDA) in progressive disease	C2
Consider lung transplantation in case of severe respiratory failure or major pulmonary hypertension	C2

### Pregnancy

There are only a few reports about pregnancy and LCH with worsening to no change of clinical symptoms, but even improvement was observed. Deterioration was mainly related to diabetes insipidus. It is unclear if worsening or onset of DI during pregnancy is really caused by LCH. This may also be observed in women not suffering from a histiocytic disorder and is caused by an accelerated degradation of vasopressin through placental enzyme vasopressinase [72].

It is unpredictable if and in which way pregnancy may influence the course of LCH. The scant literature suggests there is no adverse impact of LCH on pregnancy or birth, with exception of need for cesarean section in selected cases [73,74] (Grade C2).

### Follow up

LCH may reactivate and lead to chronic local symptoms or induce organ dysfunction. Rarely LCH is associated with malignant tumors. Therefore, follow-up investigations of disease and monitoring of functional impairments are necessary.

Restaging every 2-3 months is standard. Follow-up intervals depend on the primary extent and activity of disease within 3 to 12 months (see Table 9). In case of affirmed reactivation, clinical evaluation should include all investigations listed above (Grade D2).

**Table 9:** Recommendations for follow-up

**Test**	**Frequency**	**Grade**
**SS-LCH And No Disease Activity**		
History (especially of thirst, polyuria, cough, dyspnea, bone pain, skin changes, neurological symptoms)	• Every clinic visit	D2
Clinical assessment, blood count and blood chemistry (as described in baseline diagnostics), ultrasound	• End of therapy	D2
	• every 6 month (for the next 2 years)	
	• then once a year (for at least 3 years)	
Chest XR	• annually (for at least 3 years)	D2
**After MS-LCH And With No Disease Activity**		
History (especially of thirst, polyuria, cough, dyspnea, bone pain, skin changes, neurological symptoms)	• Every clinic visit	D2
Clinical assessment, blood count and blood chemistry (as described in baseline diagnostics), ultrasound	• End of therapy	D2
	• every 3 month (for the next 2 years)	
	• every 6 month (for the next 3 years)	
	• then once a year (for at least 5 years)	
Chest XR	• annually (for at least 3 years)	D2
TSH, freeT4	• Once a year (until end of routinely follow up)	D2
**Patients With Active Disease**		
Diagnostic procedures are depending on the site of organ involvement	Frequency is depending on rates and velocity of recurrences	D2
**Patients With pLCH**		
History (in case of non-pulmonary symptoms: look for MS LCH, see Table 4)	• Every clinic visit	D2
Diagnostic procedures are depending on symptoms und course of PLCH (baseline: Chest X-ray, lung function (+DCLO)	• End of therapy	D2
	• every 6 month (for the next 2 years)	
	• then once a year (for at least 5 years)	

## Competing interest

The authors declare that they have no competing interests.

## Authors’ contributions

Based on the available literature up to December 2012, recommendations were established, drafts were commented by the entire group, and redrafted by the executive editor. All authors read and approved the final manuscript.

## References

[B1] Arico M Langerhans cell histiocytosis in adults. Report from the International Registry of the Histiocyte Society Eur J Cancer 2003 39 16 2341 2348 14556926 10.1016/s0959-8049(03)00672-5

[B2] Willman CL Langerhans'-cell histiocytosis (histiocytosis X)–a clonal proliferative disease N Engl J Med 1994 331 3 154 160 8008029 10.1056/NEJM199407213310303

[B3] Yousem SA Pulmonary Langerhans' cell histiocytosis: molecular analysis of clonality Am J Surg Pathol 2001 25 5 630 636 11342775 10.1097/00000478-200105000-00010

[B4] Badalian-Very G Recurrent BRAF mutations in Langerhans cell histiocytosis Blood 2010 116 11 1919 1923 20519626 10.1182/blood-2010-04-279083 PMC3173987

[B5] Badalian-Very G *Pathogenesis of Langerhans Cell Histiocytosis* Annu Rev Pathol 2013 24 8 1 20 22906202 10.1146/annurev-pathol-020712-163959

[B6] Egeler RM Association of Langerhans cell histiocytosis with malignant neoplasms Cancer 1993 71 3 865 873 8431870 10.1002/1097-0142(19930201)71:3<865::aid-cncr2820710334>3.0.co;2-0

[B7] Lee JS Langerhans cell sarcoma arising from Langerhans cell histiocytosis: a case report J Korean Med Sci 2006 21 3 577 580 16778410 10.3346/jkms.2006.21.3.577 PMC2729972

[B8] Lau SK Chu PG Weiss LM Immunohistochemical expression of Langerin in Langerhans cell histiocytosis and non-Langerhans cell histiocytic disorders Am J Surg Pathol 2008 32 4 615 619 18277880 10.1097/PAS.0b013e31815b212b

[B9] Swerdlow SHC International Agency for Research on, Cancer and O. World Health: WHO classification of tumours of haematopoietic and lymphoid tissues 2008 International Agency for Research on Cancer (IARC)

[B10] Valladeau J Langerin, a novel C-type lectin specific to Langerhans cells, is an endocytic receptor that induces the formation of Birbeck granules Immunity 2000 12 1 71 81 10661407 10.1016/s1074-7613(00)80160-0

[B11] Minkov M Grois N McClain K Nanduri V Rodriguez-Galindo C Simonitsch-Klupp I Visser J Weitzmann S Whitlock J Windebank K Langerhans Cell Histiocytosis - Histiocyte Society Evaluation and Treatment Guidelines 2009 cited; Available from: http://www.histiocytesociety.org/document.doc?id=290

[B12] Arico M Familial clustering of Langerhans cell histiocytosis Br J Haematol 1999 107 4 883 888 10606898 10.1046/j.1365-2141.1999.01777.x

[B13] McClain K Bone and Soft Tissue Involvement - Oral Presentation at the Annual Meeting of the Histiocyte Society, Vienna 2011

[B14] Phillips M Comparison of FDG-PET scans to conventional radiography and bone scans in management of Langerhans cell histiocytosis Pediatr Blood Cancer 2009 52 1 97 101 18951435 10.1002/pbc.21782

[B15] Szturz P [Lymphoma-like course in aggressive adult multisystem Langerhans cell histiocytosis and the benefit of PET/CT imaging in evaluation of diffuse metabolic activity of lung parenchyma] Vnitr Lek 2010 56 11 1177 1193 21250497

[B16] Teng CL Rapidly fatal Langerhans' cell histiocytosis in an adult J Formos Med Assoc 2005 104 12 955 959 16607456

[B17] Grois N Risk factors for diabetes insipidus in langerhans cell histiocytosis Pediatr Blood Cancer 2006 46 2 228 233 16047354 10.1002/pbc.20425

[B18] Kaltsas GA Hypothalamo-pituitary abnormalities in adult patients with langerhans cell histiocytosis: clinical, endocrinological, and radiological features and response to treatment J Clin Endocrinol Metab 2000 85 4 1370 1376 10770168 10.1210/jcem.85.4.6501

[B19] Prosch H Central diabetes insipidus as presenting symptom of Langerhans cell histiocytosis Pediatr Blood Cancer 2004 43 5 594 599 15382278 10.1002/pbc.20102

[B20] Makras P Endocrine manifestations in Langerhans cell histiocytosis Trends Endocrinol Metab 2007 18 6 252 257 17600725 10.1016/j.tem.2007.06.003

[B21] Amato MC Endocrine disorders in pediatric - onset Langerhans Cell Histiocytosis Horm Metab Res 2006 38 11 746 751 17111302 10.1055/s-2006-955086

[B22] Makras P Evolving radiological features of hypothalamo-pituitary lesions in adult patients with Langerhans cell histiocytosis (LCH) Neuroradiology 2006 48 1 37 44 16292545 10.1007/s00234-005-0011-x

[B23] Donadieu J Incidence of growth hormone deficiency in pediatric-onset Langerhans cell histiocytosis: efficacy and safety of growth hormone treatment J Clin Endocrinol Metab 2004 89 2 604 609 14764769 10.1210/jc.2003-030907

[B24] Alexandraki KI Cardiovascular risk factors in adult patients with multisystem Langerhans-cell histiocytosis: evidence of glucose metabolism abnormalities QJM 2008 101 1 31 40 18160417 10.1093/qjmed/hcm118

[B25] Makras P *Reduced bone mineral density in adult patients with Langerhans cell histiocytosis* Pediatr Blood Cancer 2012 58 5 819 822 21548013 10.1002/pbc.23166

[B26] Caputo R A Text Atlas of Histiocytic Syndromes 1998 Informa HealthCare

[B27] Singhi AD Montgomery EA Gastrointestinal tract langerhans cell histiocytosis: A clinicopathologic study of 12 patients Am J Surg Pathol 2011 35 2 305 310 21263252 10.1097/PAS.0b013e31820654e4

[B28] Yasko AW Percutaneous techniques for the diagnosis and treatment of localized Langerhans-cell histiocytosis (eosinophilic granuloma of bone) J Bone Joint Surg Am 1998 80 2 219 228 9486728 10.2106/00004623-199802000-00009

[B29] Lo WC Isolated adult Langerhans' cell histiocytosis in cervical lymph nodes: should it be treated? J Laryngol Otol 2009 123 9 1055 1057 19046468 10.1017/S0022215108004155

[B30] Hoeger PH Long term follow up of topical mustine treatment for cutaneous langerhans cell histiocytosis Arch Dis Child 2000 82 6 483 487 10833183 10.1136/adc.82.6.483 PMC1718363

[B31] Sakai H Satisfactory remission achieved by PUVA therapy in Langerhans cell hisiocytosis in an elderly patient J Dermatol 1996 23 1 42 46 8720257 10.1111/j.1346-8138.1996.tb03966.x

[B32] Imafuku S Cutaneous Langerhans cell histiocytosis in an elderly man successfully treated with narrowband ultraviolet B Br J Dermatol 2007 157 6 1277 1279 17916205 10.1111/j.1365-2133.2007.08204.x

[B33] Sander CS Kaatz M Elsner P Successful treatment of cutaneous langerhans cell histiocytosis with thalidomide Dermatology 2004 208 2 149 152 15057007 10.1159/000076491

[B34] McClain KL Kozinetz CA A phase II trial using thalidomide for Langerhans cell histiocytosis Pediatr Blood Cancer 2007 48 1 44 49 16333818 10.1002/pbc.20578

[B35] Chu A Dermatological Aspects and Presentation of an Adult Clinic - Oral Presentation at the Annual Meeting of the Histiocyte Society, Vienna 2011

[B36] Steen AE Successful treatment of cutaneous Langerhans cell histiocytosis with low-dose methotrexate Br J Dermatol 2001 145 1 137 140 11453923 10.1046/j.1365-2133.2001.04298.x

[B37] Cantu MA Optimal therapy for adults with Langerhans cell histiocytosis bone lesions PLoS One 2012 7 8 e43257 22916233 10.1371/journal.pone.0043257 PMC3419729

[B38] Derenzini E MACOP-B regimen in the treatment of adult Langerhans cell histiocytosis: experience on seven patients Ann Oncol 2010 21 6 1173 1178 19861578 10.1093/annonc/mdp455

[B39] Montella L Zoledronic acid in treatment of bone lesions by Langerhans cell histiocytosis J Bone Miner Metab 2009 27 1 110 113 19018458 10.1007/s00774-008-0001-2

[B40] Reichle A Anti-inflammatory and angiostatic therapy in chemorefractory multisystem Langerhans' cell histiocytosis of adults Br J Haematol 2005 128 5 730 732 15725096 10.1111/j.1365-2141.2004.05359.x

[B41] McClain KL Drug therapy for the treatment of Langerhans cell histiocytosis Expert Opin Pharmacother 2005 6 14 2435 2441 16259575 10.1517/14656566.6.14.2435

[B42] Montella L Insabato L Palmieri G Imatinib mesylate for cerebral Langerhans'-cell histiocytosis N Engl J Med 2004 351 10 1034 1035 15342818 10.1056/NEJM200409023511022

[B43] Janku F Response of histiocytoses to imatinib mesylate: fire to ashes J Clin Oncol 2010 28 31 e633 e636 20733125 10.1200/JCO.2010.29.9073

[B44] Ingram W Reduced-intensity conditioned allogeneic haematopoietic transplantation in an adult with Langerhans' cell histiocytosis and thrombocytopenia with absent radii Bone Marrow Transplant 2006 37 7 713 715 16489360 10.1038/sj.bmt.1705300

[B45] Xicoy B [Sustained remission in an adult patient with Langerhans cell histiocytosis following T-cell depleted allogenic cell transplantation] Med Clin (Barc) 2006 127 18 716 17169302 10.1157/13095100

[B46] Rodriguez-Galindo C Clofarabine in refractory Langerhans cell histiocytosis Pediatr Blood Cancer 2008 51 5 703 706 18623218 10.1002/pbc.21668

[B47] Saven A Burian C Cladribine activity in adult langerhans-cell histiocytosis Blood 1999 93 12 4125 4130 10361109

[B48] Atalar B Adult langerhans cell histiocytosis of bones : a rare cancer network study Acta Orthop Belg 2010 76 5 663 668 21138223

[B49] Gaundong Mbethe GL [Multifocal Langerhans cell histiocytosis of bone: indications for radiotherapy] Cancer Radiother 2010 14 8 759 762 20674449 10.1016/j.canrad.2010.03.018

[B50] Brady LW Olschewski T, Seegenschmiedt MH, Micke O Langerhans Cell Histiocytosis Langerhans Cell Histiocytosis. 2008 Springer Verlag 397 423

[B51] Greenberger JS Radiation therapy in patients with histiocytosis: management of diabetes insipidus and bone lesions Int J Radiat Oncol Biol Phys 1979 5 10 1749 1755 316811 10.1016/0360-3016(79)90556-x

[B52] Heyd R Radiotherapy in Langerhans-cell histiocytosis. 2 case reports and review of the literature Rontgenpraxis 2000 53 2 51 61 10994366

[B53] Micke O Seegenschmiedt MH Consensus guidelines for radiation therapy of benign diseases: a multicenter approach in Germany Int J Radiat Oncol Biol Phys 2002 52 2 496 513 11872298 10.1016/s0360-3016(01)01814-4

[B54] Cassady JR Current role of radiation therapy in the management of histiocytosis-X Hematol Oncol Clin North Am 1987 1 1 123 129 3312144

[B55] Grois N Neuropathology of CNS disease in Langerhans cell histiocytosis Brain 2005 128 Pt 4 829 838 15705614 10.1093/brain/awh403

[B56] Grois N Central nervous system disease in Langerhans cell histiocytosis J Pediatr 2010 156 6 873 881 881 e1 20434166 10.1016/j.jpeds.2010.03.001

[B57] Idbaih A Retinoic acid therapy in "degenerative-like" neuro-langerhans cell histiocytosis: a prospective pilot study Pediatr Blood Cancer 2004 43 1 55 58 15170890 10.1002/pbc.20040

[B58] Imashuku S Treatment of neurodegenerative CNS disease in Langerhans cell histiocytosis with a combination of intravenous immunoglobulin and chemotherapy Pediatr Blood Cancer 2008 50 2 308 311 17458874 10.1002/pbc.21259

[B59] Chohan G **Langerhans cell histiocytosis with refractory central nervous system involvement responsive to infliximab** J Neurol Neurosurg Psychiatry 2012 83 5 573 575 21856693 10.1136/jnnp-2011-300575

[B60] Allen CE Neurodegenerative central nervous system Langerhans cell histiocytosis and coincident hydrocephalus treated with vincristine/cytosine arabinoside Pediatr Blood Cancer 2010 54 3 416 423 19908293 10.1002/pbc.22326 PMC3444163

[B61] Allen CE Personal communication about Neurodegenerative central nervous system Langerhans cell histiocytosis and coincident hydrocephalus treated with vincristine/cytosine arabinoside 2010 10.1002/pbc.22326 PMC3444163 19908293

[B62] Tazi A Adult pulmonary Langerhans' cell histiocytosis Eur Respir J 2006 27 6 1272 1285 16772390 10.1183/09031936.06.00024004

[B63] Watanabe R Clinico-epidemiological features of pulmonary histiocytosis X Intern Med 2001 40 10 998 1003 11688843 10.2169/internalmedicine.40.998

[B64] Vassallo R Clinical outcomes of pulmonary Langerhans'-cell histiocytosis in adults N Engl J Med 2002 346 7 484 490 11844849 10.1056/NEJMoa012087

[B65] Tazi A Marc K Dominique S de Bazelaire C Crestani B Chinet T Israel-Biet D Cadranel J Frija J Lorillon G Valeyre D Chevret S **Serial CT and lung function testing in pulmonary Langerhans cell histiocytosis** Eur Respir J 2012 40 4 905 912 22441752 10.1183/09031936.00210711

[B66] Auerswald U Barth J Magnussen H Value of CD-1-positive cells in bronchoalveolar lavage fluid for the diagnosis of pulmonary histiocytosis X Lung 1991 169 6 305 309 1758200 10.1007/BF02714167

[B67] Lepavec J Lorillon G Jaïs X Tcherakian C Feuillet S Dorfmüller P Simonneau G Humbert M Tazi A Pulmonary Langerhans Cell Histiocytosis associated pulmonary hypertension: clinical characteristics and impact of pulmonary arterial hypertension therapies Chest 2012 10.1378/chest.11-2490 22459770

[B68] Lazor R Progressive diffuse pulmonary Langerhans cell histiocytosis improved by cladribine chemotherapy Thorax 2009 64 3 274 5 19252036 10.1136/thx.2008.108944

[B69] Lorillon G Cladribine is effective against cystic pulmonary Langerhans cell histiocytosis Am J Respir Crit Care Med 2012 186 9 930 2 23118088 10.1164/ajrccm.186.9.930

[B70] Mendez JL Pneumothorax in pulmonary Langerhans cell histiocytosis Chest 2004 125 3 1028 32 15006964 10.1378/chest.125.3.1028

[B71] Dauriat G Lung transplantation for pulmonary langerhans' cell histiocytosis: a multicenter analysis Transplantation 2006 81 5 746 50 16534477 10.1097/01.tp.0000200304.64613.af

[B72] Ananthakrishnan S Diabetes insipidus in pregnancy: etiology, evaluation, and management Endocr Pract 2009 15 4 377 82 19454377 10.4158/EP09090.RA

[B73] DiMaggio LA Lippes HA Lee RV Histiocytosis X and pregnancy Obstet Gynecol 1995 85 5 Pt 2 806 9 7724119 10.1016/0029-7844(94)00404-2

[B74] Sharma R Maplethorpe R Wilson G Effect of pregnancy on lung function in adult pulmonary Langerhans cell histiocytosis J Matern Fetal Neonatal Med 2006 19 1 67 8 16492595 10.1080/14767050500333835

